# *EGFR*-Mutated Lung Adenocarcinoma Successfully Treated With Osimertinib After Spontaneous Transformation to SCLC and Adenocarcinoma With Neuroendocrine Differentiation: Case Report

**DOI:** 10.1016/j.jtocrr.2021.100264

**Published:** 2021-12-04

**Authors:** Sho Takuma, Yusuke Inoue, Masato Karayama, Kazuo Tsuchiya, Hiroe Tsukui, Hironao Hozumi, Yuzo Suzuki, Kazuki Furuhashi, Noriyuki Enomoto, Tomoyuki Fujisawa, Yutaro Nakamura, Naoki Inui, Takafumi Suda

**Affiliations:** a Second Division, Department of Internal Medicine, Hamamatsu University School of Medicine, Hamamatsu, Japan; b Department of Clinical Pharmacology and Therapeutics, Hamamatsu University School of Medicine, Hamamatsu, Japan; c Department of Clinical Oncology, Hamamatsu University School of Medicine, Hamamatsu, Japan; d Department of Diagnostic Pathology, Hamamatsu University School of Medicine, Hamamatsu, Japan; e Department of Laboratory Medicine, Hamamatsu University School of Medicine, Hamamatsu, Japan

**Keywords:** Spontaneous transformation, Neuroendocrine, EGFR, Small cell lung cancer, Case report

## Abstract

Lineage transformation from lung adenocarcinoma (LUAD) to SCLC is associated with resistance to EGFR tyrosine kinase inhibitors. In addition to loss of p53 and RB, transformed SCLCs are usually not dependent on EGFR signaling, which renders the tumors unresponsive to EGFR tyrosine kinase inhibitors. Here, we present a case of spontaneous transformation from *EGFR*-mutant LUAD with loss of p53 and RB to EGFR expression-positive SCLC and neuroendocrine-differentiated LUAD, which was successfully treated with osimertinib.

## Introduction

Lineage transformation from lung adenocarcinoma (LUAD) to SCLC is an important nongenetic resistance mechanism against EGFR tyrosine kinase inhibitors (TKIs) in *EGFR*-mutant LUAD. *EGFR*-mutant LUAD is known to be at unique risk of SCLC transformation, particularly when EGFR signaling is abolished by inhibitors and p53 and RB are inactivated.^1^^,^^2^ Transformed SCLC is characterized by the loss of EGFR expression despite original *EGFR* mutations being retained,^3^ as activation of the downstream MAPK pathway is incompatible for SCLC through the suppression of neuroendocrine differentiation.^4^ Therefore, transformed *EGFR*-mutant SCLC is usually no longer dependent on EGFR signaling and is refractory to EGFR TKIs.^3^ Here, we report a case of spontaneously transformed *EGFR*-mutant SCLC and neuroendocrine-differentiated LUAD successfully treated with osimertinib.

## Case Presentation

**Figure 1:**
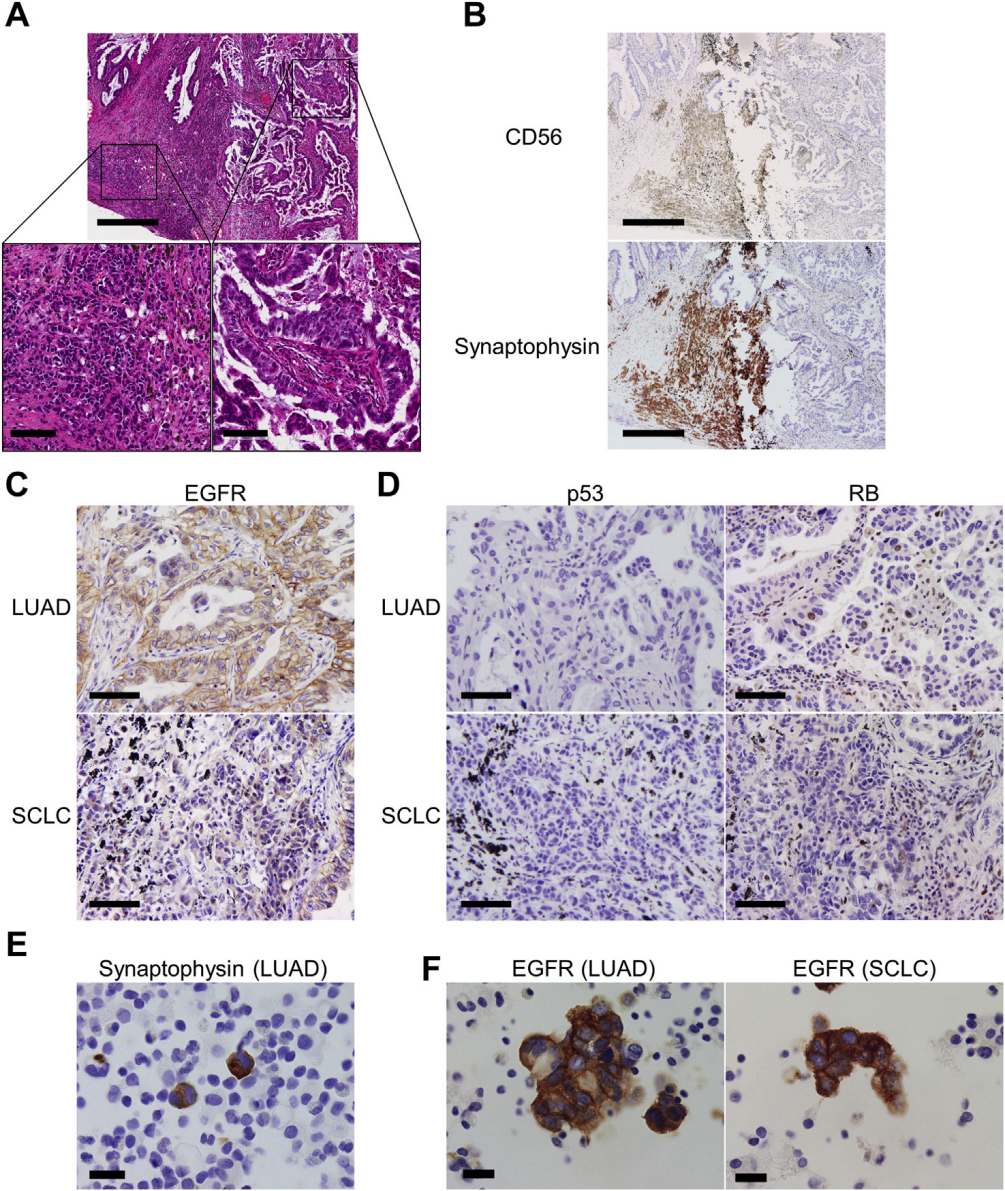
Histologic and cytologic evaluation of the tumor cells. (*A*) H&E staining images of the resected metastatic lung tumor at low power (upper) and high power revealing the LUAD (lower, right) and SCLC (lower, left) components. The tumor mostly consists of papillary-dominant LUAD. The 1.5-mm component of SCLC cells with high nuclear-cytoplasmic ratio is identified. (*B–D*) IHC sections of LUAD and SCLC cells in the resected lung tumors for (*B*) CD56 and synaptophysin, (*C*) EGFR, and (*D*) p53 and RB. (*E*, *F*) Cell block immunocytochemistry sections of the pleural fluid specimen at the time of recurrence for (*E*) synaptophysin in LUAD cells with relatively abundant cytoplasm and (*F*) EGFR in LUAD and SCLC cells. Scale bars in sections: (*A*, upper) 500 μm; (*A*, lower) 100 μm; (*B*) 500 μm; (*C* and *D*) 100 μm; and (*E* and *F*) 20 μm. H&E, hematoxylin and eosin; IHC, immunohistochemistry; LUAD, lung adenocarcinoma.

**Figure 2:**
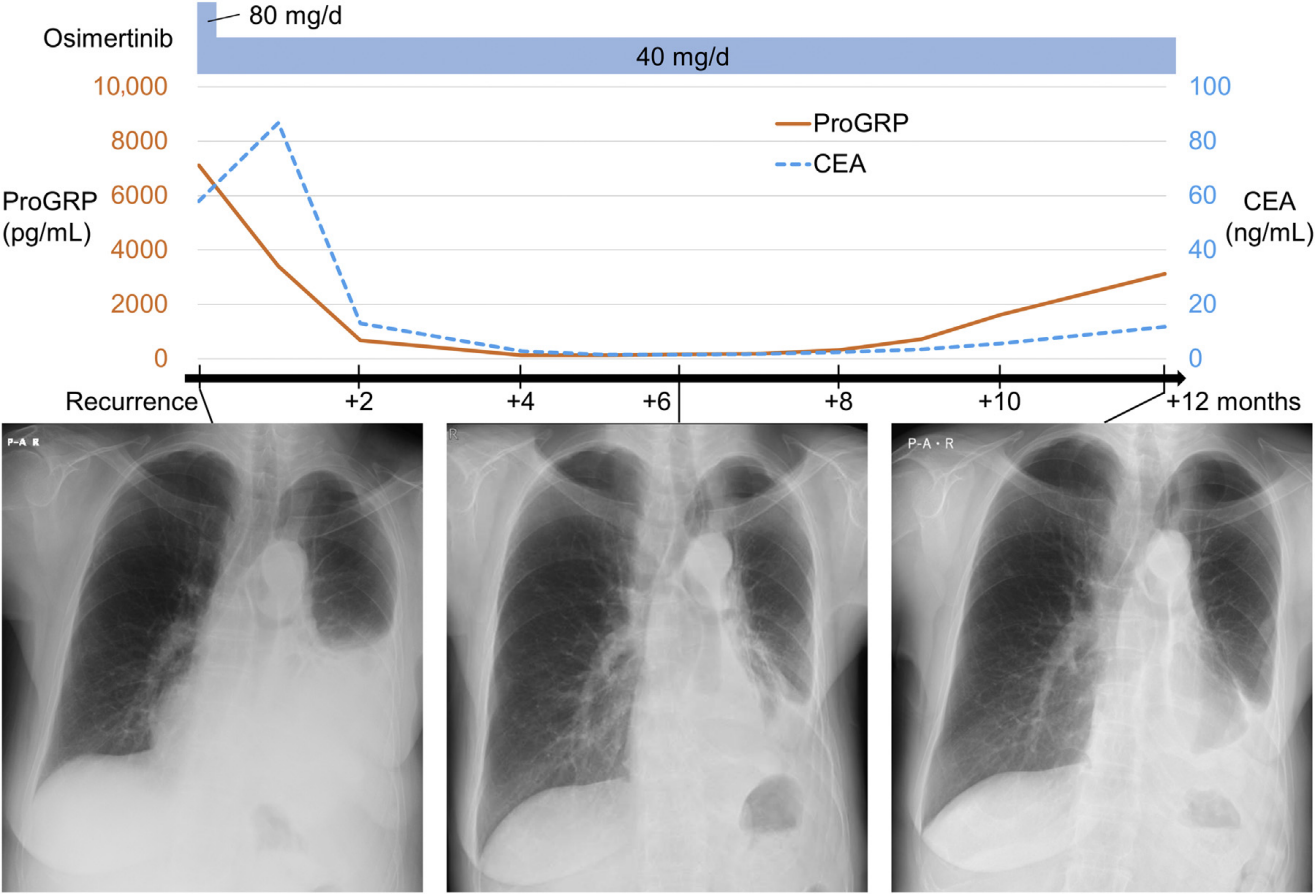
Timeline of the clinical, tumor markers, and radiological course and treatment from the diagnosis of disease recurrence. CEA, carcinoembryonic antigen; ProGRP, progastrin-releasing peptide.

A 74-year-old woman who had never smoked was found on chest computed tomography to have a mass and a small nodule in the upper lobe of her left lung. Her serum carcinoembryonic antigen (CEA) and progastrin-releasing peptide (ProGRP) levels were elevated at 121 ng/mL and 97.5 pg/mL, respectively. She was given a diagnosis of LUAD on the basis of histologic examination after a transbronchial biopsy and underwent left upper lobectomy. Histologic examination of the resected tumors revealed that the primary tumor was papillary-dominant LUAD but the metastatic tumor (11 mm in length) contained a focal SCLC component with a diameter of 1.5 mm (Fig. 1*A*). LUAD cells at the primary site invaded to the visceral pleural surface and SCLC cells at the metastatic site invaded beyond the elastic layer (pl2; pT3N0M0; stage IIB). Although the LUAD cells were negative for CD56 and synaptophysin, SCLC cells were positive for both markers (Fig. 1*B*). SCLC cells had a Ki-67 labeling index of 80%. *EGFR* genotyping performed using primary LUAD tumor cells revealed an *EGFR* exon 19 deletion (Del19) mutation. Of note, Del19 was also detected in the microdissected SCLC cells at the metastatic site. EGFR as assessed by immunohistochemistry using the 31G7 antibody (Nichirei) was strongly expressed in LUAD cells and weakly expressed in SCLC cells (Fig. 1*C*). Expression of p53 (clone DO-7; Dako) was not observed in either component cell (Fig. 1*D*). RB (clone G3-245; BD Pharmingen) was expressed only in a few of the LUAD cells but not expressed in the SCLC cells (Fig. 1*D*). On the basis of the postoperative pathologic diagnosis of combined SCLC with adenocarcinoma or LUAD with transformed SCLC, postoperative adjuvant chemotherapy with carboplatin and etoposide was administered; however, chemotherapy was terminated after one course owing to febrile neutropenia and the patient’s decline. After one year from the surgery, she presented with dyspnea owing to massive left pleural effusion. Along with serum CEA (58 ng/mL), serum ProGRP level was markedly elevated at 7116 pg/mL. Pleural fluid cytology identified both LUAD and SCLC cells. Both component cells were positive for CD56 and synaptophysin (Fig. 1*E*), indicating even adenocarcinoma cells had neuroendocrine transdifferentiation. Both LUAD and SCLC cells were again positive for EGFR (Fig. 1*F*) but negative for p53 and RB. In addition, *EGFR* genotyping using a pleural fluid cell block identified an identical Del19. Treatment with osimertinib (80 mg/d) was initiated. Although osimertinib was reduced to 40 mg/d owing to the development of leg edema on day 10, pleural effusion and both tumor markers continued to be markedly decreased. Nevertheless, tumor markers became elevated again after 8 months and fluorodeoxyglucose-positron emission tomography revealed a left pleural nodule with fluorodeoxyglucose accumulation, which was judged to be a disease progression. At the time of writing, osimertinib treatment was continued beyond progressive disease, with the pleural effusion remaining controlled for a total of 12 months from the initiation of the treatment (Fig. 2).

## Discussion

Here, we describe a patient with a Del19 mutation whose primary tumor contained LUAD only and whose metastatic tumor contained LUAD along with a minute SCLC component. Pleural effusion at the time of recurrence was composed of both SCLC and neuroendocrine-differentiated LUAD cells. Although typical SCLC cells and transformed *EGFR*-mutant SCLC cells have been reported to lack EGFR expression,^3^^,^^4^ SCLC cells in this patient were positive for EGFR. Moreover, both LUAD and SCLC cells were negative for p53 and RB. In addition, the patient had no history of smoking, despite SCLC carcinogenesis being strongly associated with smoking and SCLC being exceedingly rare in individuals who have never smoked. Furthermore, the identical Del19 was detected in the LUAD and SCLC cells. These observations collectively suggested that SCLC cells in our case were transformed from LUAD cells rather than this representing a combination of independent SCLC and LUAD cells. Spontaneous SCLC transformation without the therapeutic positive selection by EGFR TKIs is experimentally supported by the finding that type 2 alveolar cells with biallelic loss of *TP53* and *RB1* can give rise to SCLC and LUAD.^5^

At the time of recurrence, even the remaining LUAD cells had neuroendocrine differentiation, which was presumably acquired during the dormant period after surgery because initial adenocarcinoma cells had lacked expression of neuroendocrine markers. This suggests that the tumor cells in this patient were susceptible to neuroendocrine transformation, partly owing to the loss of p53 and RB; a clone branched off from the founder cells initially and transformed into SCLC identified in the resected specimen; another clone branched off later after surgery and acquired neuroendocrine features with persistent LUAD cytology.

**Figure 3:**
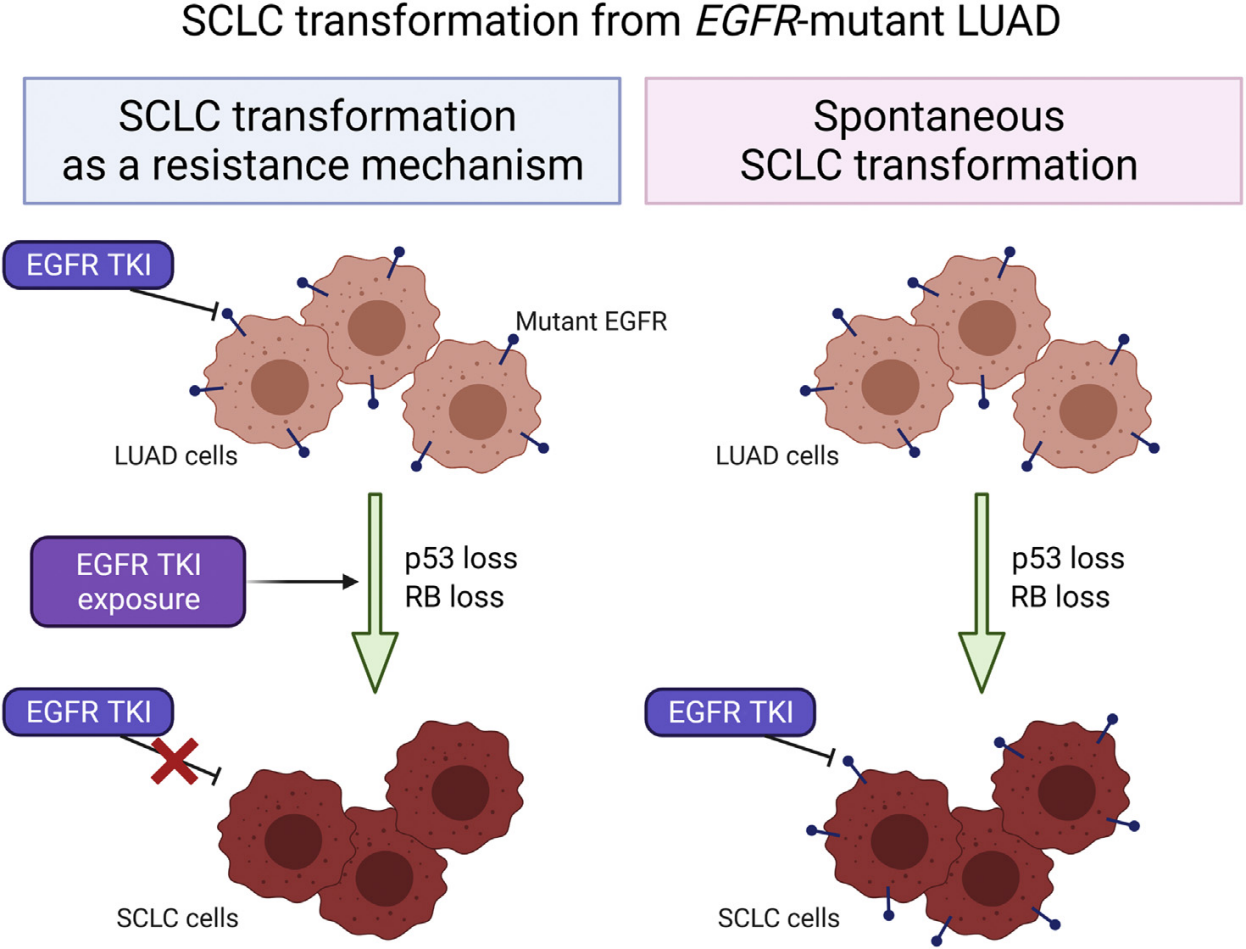
Schematic representation of *EGFR-*mutant transformed SCLC with or without exposure to EGFR TKIs during the evolution. The present case is represented on the right side. Illustration was created with BioRender.com. LUAD, lung adenocarcinoma; TKIs, tyrosine kinase inhibitors.

SCLC transformation from *EGFR*-mutant LUAD has been reported to be associated with resistance to EGFR TKIs.^1^^,^^2^^,^^4^ Nevertheless, it remains unknown whether EGFR TKIs could be used for EGFR TKI-naive transformed SCLC with *EGFR* mutations because of the rarity of these cases. Persistence of an *EGFR* mutation and dependency on EGFR signaling should be necessary for achieving efficacy of EGFR TKIs. In this case, the SCLC component-specific genotyping of *EGFR* at the time of recurrence could not be performed owing to the nature of the cell block. Instead, we observed that both LUAD and SCLC cells were strongly positive for EGFR expression (Fig. 1*F*). It would account for the achieved long-lasting disease control by osimertinib of both LUAD and SCLC cells which was supported by the marked decrease in both tumor markers CEA and ProGRP likely representing the disease burden of LUAD and SCLC, respectively (Fig. 3). The present case suggests that EGFR TKIs could be a therapeutic option for spontaneously transformed SCLC carrying *EGFR* mutations with functional EGFR.

## Conclusion

Identifying the p53 and RB status of patients might be important to monitor those with *EGFR*-mutant LUAD for possible transformation to the neuroendocrine lineage. Combined assessment of *EGFR* mutation status and EGFR pathway activity would predict the efficacy of EGFR TKIs in patients with *EGFR*-mutated spontaneously transformed SCLC.

## CRediT Authorship Contribution Statement

**Sho Takuma:** Conceptualization, Data curation, Visualization, Writing—original draft.

**Yusuke Inoue:** Conceptualization, Visualization, Writing—original draft, Writing—review and editing, Supervision.

**Masato Karayama, Kazuo Tsuchiya, Hiroe Tsukui, Hironao Hozumi, Yuzo Suzuki, Kazuki Furuhashi, Noriyuki Enomoto, Tomoyuki Fujisawa, Yutaro Nakamura, Naoki Inui, Takafumi Suda:** Validation, Writing—review and editing, Supervision.

## References

[1] Ferrer L., Levra M.J., Brevet M. (2019). A brief report of transformation from NSCLC to SCLC: molecular and therapeutic characteristics. J Thorac Oncol.

[2] Offin M., Chan J.M., Tenet M. (2019). Concurrent *RB1* and *TP53* alterations define a subset of *EGFR*-mutant lung cancers at risk for histologic transformation and inferior clinical outcomes. J Thorac Oncol.

[3] Niederst M.J., Sequist L.V., Poirier J.T. (2015). RB loss in resistant *EGFR* mutant lung adenocarcinomas that transform to small-cell lung cancer. Nat Commun.

[4] Inoue Y., Nikolic A., Farnsworth D. (2021). Extracellular signal-regulated kinase mediates chromatin rewiring and lineage transformation in lung cancer. Elife.

[5] Sutherland K.D., Proost N., Brouns I., Adriaensen D., Song J.Y., Berns A. (2011). Cell of origin of small cell lung cancer: inactivation of *Trp53* and *Rb1* in distinct cell types of adult mouse lung. Cancer Cell.

